# The Effect of Molecular Crowding on the Stability of Human c-MYC Promoter Sequence I-Motif at Neutral pH

**DOI:** 10.3390/molecules181012751

**Published:** 2013-10-15

**Authors:** Jingjing Cui, Phillip Waltman, Vu H. Le, Edwin A. Lewis

**Affiliations:** Department of Chemistry, Mississippi State University, Mississippi State, MS 39762, USA

**Keywords:** i-motif, stability, neutral pH, molecular crowding, excluded volume effect

## Abstract

We have previously shown that c-MYC promoter sequences can form stable i-motifs in acidic solution (pH 4.5–5.5). In terms of drug targeting, the question is whether c-MYC promoter sequence i-motifs will exist in the nucleus at neutral pH. In this work, we have investigated the stability of a mutant c-MYC i-motif in solutions containing a molecular crowding agent. The crowded nuclear environment was modeled by the addition of up to 40% w/w polyethylene glycols having molecular weights up to 12,000 g/mol. CD and DSC were used to establish the presence and stability of c-MYC i-motifs in buffer solutions over the pH range 4 to 7. We have shown that the c-MYC i-motif can exist as a stable structure at pH values as high as 6.7 in crowded solutions. Generic dielectric constant effects, e.g., a shift in the p*K*a of cytosine by more than 2 units (e.g., 4.8 to 7.0), or the formation of non-specific PEG/DNA complexes appear to contribute insignificantly to i-motif stabilization. Molecular crowding, largely an excluded volume effect of added PEG, having a molecular weight in excess of 1,000 g/mol, appears to be responsible for stabilizing the more compact i-motif over the random coil at higher pH values.

## 1. Introduction

Regulation of the expression of oncogenes such as K-Ras [1], Bcl-2 [2] and/or c-MYC [3] may provide avenues for the control of many important biological processes such as cell replication, translation, apoptosis, *etc.* Our research group in particular has published G-quadruplex studies on these oncogenes which have demonstrated G-quadruplex- and i-motif-forming sequences in their promoter regions. Located on the human chromosome 8, the c-MYC gene is responsible for encoding a transcription factor that can regulate many genes that can further affect cell function, cell growth and cell apoptosis [4]. The c-MYC gene is associated with a wide variety of cancers exhibiting abnormally high levels of c-MYC expression [5,6]. It is well known that the degree of c-MYC expression is a controlling factor in apoptosis: reducing the expression of the c-MYC gene directly induces apoptosis [7,8]. The c-MYC transcription machinery involves multiple components including several promoters, proteins, and nuclease hypersensitive elements. One of the nuclease hypersensitive elements, NHE III_1_, is believed to control over 90% of the c-MYC gene transcription [9,10,11,12,13,14,15]. The NHE III_1_ is located ~142 to ~115 bp upstream of P1 promoter, and is capable of forming higher order DNA structures such as looped out G-quadruplexes and i-motifs due to its GC-rich rich sequence [16,17,18].

**Figure 1:**
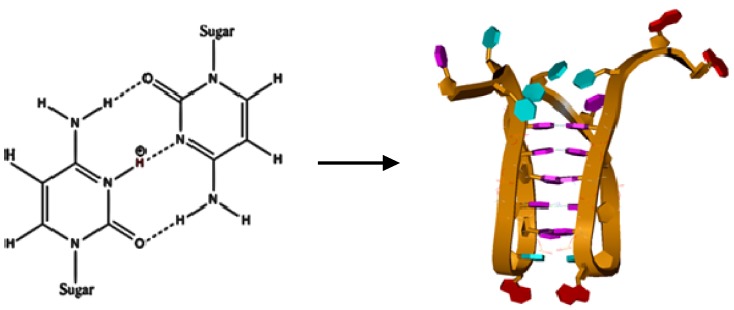
The chemical structure for a hemi-protonated Cyt-3N^+^-H/N3-Cyt base pair formed by Hoogsteen hydrogen binding between the two cytosine bases (typical at pH ≈ 5). The molecular model represents one conformer of the c-MYC promoter sequence i-motif, stabilized by five Hoogsteen hydrogen bonded cytosine pairs. The adenine purines are shown in red, the thymine pyrimidines are shown in blue, and the cytosine pyrimidines are shown in purple.

Intercalated motifs or i-motifs are formed by the unusual cytosine/cytosine base pairing shown in Figure 1 and the unusual folding of the DNA backbone to create a tetraplex DNA structure with intercalated cytosine base pairs. The cytosine base pairing is most favorable at pH values near the p*K*_a_ of cytosine N3 (p*K*_a_ ≈ 4.8, in water). In effect, the i-motif is most stable at the pH where one half of the cytosines are protonated and three H-bonds are formed between the two paired cytosines. While G-quadruplexes formed by G-rich sequences have received more attention since their discovery, studies of the i-motif formed by C-rich complementary strands have been rather limited. To date, most i-motif studies have focused on geometry, symmetry, folding topology and subsequent stability. Although most C-rich sequences (including the c-MYC promoter sequence) can form an ensemble of i-motifs depending on folding topology and the specific cytosines being paired, a typical structure for the c-MYC i-motif is shown in Figure 1.

The mutant c-MYC promoter sequence, (5′-TTTTCCTACCCTTCCTACCCTAA-3′), used in this study was designed on the basis of previous studies, to yield a simple folding topology. In fact a single melting transition is observed for this sequence in DSC experiments. Computational modeling of the intramolecular i-motifs formed by a similar mutant c-MYC 23-mer P1 promoter sequence, (5′-CTTTCCTACCCTCCCTACCCTAA-3′), yielded an ensemble of five different possible i-motif structures, of which three were observed experimentally in DSC experiments [19]. The structure shown in Figure 1 is the most stable of the five proposed c-MYC i-motifs previously reported [19]. Similar structures have been confirmed by NMR [20,21], X-ray crystallography [22,23], and FRET [24] for the human telomere i-motif. For the reasons discussed above, the typical i-motif is most stable in acidic solution (pH 4.5–5.5) [19,25,26]. The stability of the typical i-motif structure decreases when the pH of the solution increases, due to the loss of the hemi-protonated Cyt-3N^+^-H / N3-Cyt pair [24,25,26,27,28,29].

To date, most of the studies of i-motif structure and stability have been done on oligonucleotides in dilute solutions with little or no co-solute or molecular crowding agent present. However, the nuclear solution environment is anything but dilute. Macromolecules, e.g., DNA, various RNAs, and numerous proteins are present in the nucleus, with water accounting for only 60% of the solution mass in the nucleus [30,31]. The nuclear solution is more like a gel, with these macromolecules contributing to the nuclear solution properties, *i.e.*, density, viscosity, and bulk dielectric constant. The fundamental difference between smaller co-solutes and these larger macromolecules is that the polymers have much larger excluded volume effects. The excluded volume affects the chemical and biochemical reactions happening in the cell [31,32]. The crowded environment can also affect kinetics (reaction rates), thermodynamics (reaction equilibria) and obviously reduces the activity of water [33,34,35,36,37]. Considerable research has already been done on the effects of molecular crowding agents on folding dynamics of DNA or proteins, and protein-DNA interactions [38,39,40,41,42,43,44]. In particular, the use of PEG as molecular crowding agent in the studies of stability and ligand interactions of G-quadruplex has been reported widely in the literature [45,46,47,48,49]. However, there are limited studies on the influence of molecular crowding agents on the i-motif structure or stability.

The efficacy of using i-motif forming sequences (C-rich sequences), located in the NHE III_1_ P1 promoter region, as a target for drug therapeutics, would depend on the existence of a stable i-motif structure in the nucleus at or near neutral pH. If stabilization of the P1 promoter sequence i-motif can be achieved, it would be possible to target the c-MYC or other oncogene promoter sequence i-motifs with drugs designed to form stable drug/i-motif complexes and thereby down-regulate the expression of these oncogenes.

In this work, we have investigated the potential for the formation of stable c-MYC i-motif structures at physiological pH in crowded environments. The question is whether a crowded environment favors the i-motif conformation over a random coil conformation for the DNA. We attempted to mimic the crowded nuclear environment by adding as much as 40% w/w polyethylene glycol to dilute solutions of the c-MYC C-rich oligonucleotide. The effects of crowding agent concentration and molecular weight have been explored in these studies. Polyethylene glycol (PEG) concentrations were varied from 10% to 40%, and the PEG molecular weight was varied from 200 g/mol to 12,000 g/mol. We present data that show the presence of the classical i-motif structure for the c-MYC P1 promoter sequence at pH values as high as 6.7. We have also shown that PEG effects are a mixture of co-solute and dielectric constant effects in combination with excluded volume or molecular crowding effects. All of these effects are linearly dependent on the PEG concentration while the molecular crowding effect is also dependent on the PEG molecular weight. The use of PEG as a molecular crowding agent is complicated by the fact that as the PEG molecular weight is increased above 4,000 and/or the PEG concentration is increased to 30 or 40% (w/w), PEG begins to interact with DNA (and probably RNA) forming non-specific DNA/PEG complexes. However, the results of this work still point to the fact that the c-MYC and other oncogene i-motifs are stabilized in crowded solution environments and therefore should be stable at neutral pH and present in the nucleus under physiological conditions.

## 2. Results and Discussion

**Figure 2:**
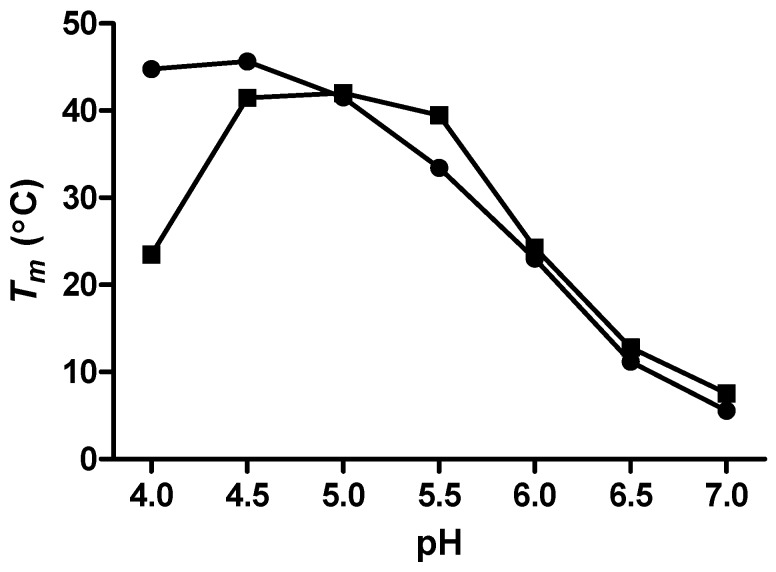
Melting temperatures, *T*_m_s, for the c-MYC mutant promoter sequence i-motif in BPES buffer (●) and with added glycerol (■) at 20% w/w.

The melting temperatures (*T_m_*s) for the mutant c-MYC P1 promoter sequence i-motifs were first determined in both BPES (0% glycerol) and 20% w/w glycerol-containing solutions having pH values of 4.0, 4.5, 5.0, 5.5, 6.0, 6.5, and 7.0, using DSC. Theses *T_m_* data are shown in Figure 2 for the unfolding of the mutant c-MYC P1 promoter i-motif at the seven specific pH values in a phosphate buffer containing 30 mM (KH_2_PO_4_, K_2_HPO_4_), 1 mM EDTA, and 100 mM KCl, BPES buffer, with and without added glycerol.

The *T_m_*s for the mutant c-MYC P1 promoter i-motif forming sequence in solutions containing 20% w/w glycerol indicate that in comparison to the *T_m_*s determined in buffer without co-solute, the i-motif structure is destabilized at pH values below 5.0, and stabilized at pH 5.5. The addition of glycerol is responsible for lowering the water activity and lowering the solvent dielectric constant. The lowering of the solvent dielectric constant is undoubtedly exerting some influence over the coulombic interactions responsible for formation of the cytosine/cytosine base pairs. In effect, we are proposing that destabilization of the i-motif at lower pH values (e.g., 4.0 and 4.5) is the result of a strengthened repulsive interaction between the two fully protonated cytosines (Cyt-H^+^/H^+^-Cyt). Alternatively, the stabilization at pH 5.5 is the result of a strengthened Cyt-H^+^/Cyt interaction between the two hemi-protonated cytosines at the higher pH.

DSC was used to determine the melting temperatures (*T_m_*s) for the c-MYC P1 promoter sequence i-motif in solutions containing PEG as the co-solute or molecular crowding agent, and at pH values of 4.0, 4.5, 5.0, 5.5, 6.0, 6.5, and 7.0. In these experiments, the added PEGs had molecular weights that varied from 200 g/mol to 4,000 g/mol. In Table 1, we have listed the measured *T_m_*s at the seven specific pH values for the i-motif in BPES buffer and the change in the *T_m_*, Δ*T_m_*, brought about by the addition of 20% PEG (PEG_200_, PEG_400_, PEG_1000_, PEG_2000_ and PEG_4000_).

**Table 1:** Change in the c-MYC i-motif melting temperature, *T_m_*, as a function of pH and 20% w/w molecular crowding agent (PEG_200_, PEG_400_, PEG_1000_, PEG_2000_, PEG_4000_, PEG_8000_).

	*T_m_* (°C)	Δ*T_m_ = T_m_(PEG) − T_m_(BPES)* ^*^					
pH	BPES	PEG_200_	PEG_400_	PEG_1000_	PEG_2000_	PEG_4000_	PEG_8000_
4.0	44.8 ± 0.2	−8.8	−4.2	−0.6	−1.6	3.9	-
4.5	45.6 ± 0.2	−0.7	1.0	3.2	4.5	5.9	5.3
5.0	41.5 ± 0.2	1.0	4.4	7.9	6.8	7.9	8.8
5.5	33.4 ± 0.2	5.6	9.3	14.7	13.1	14.3	12.8
6.0	23.0 ± 0.2	5.6	11.7	13.3	15.8	13.9	9.5
6.5	11.2 ± 0.2 ^‡^	5.2	7.0	6.4	8.8	9.7	6.5 ^‡^
7.0	5.6 ± 0.2	1.8	1.0	0.0	1.7	5.2	-

* The uncertainty in Δ*T_m_* is approximately ± 0.4 °C. ^‡^ *T_m_* and Δ*T_m_* shown in red are for i-motifs that melt below 20 °C.

The stabilization of the i-motif by the addition of PEG is evident from the increases in *T_m_* values at all pH values above 4.5. Obviously the *T_m_* is dependent on pH in all solutions and on the PEG molecular weight. In the mid pH range, (*i.e.*, 5.0 < pH < 6.0), the *T_m_* measured is attributed to the unfolding of the i-motif while at higher pH values (*i.e.*, pH > 6.0) the DNA samples that undergo thermal unfolding are not all classical i-motifs (see CD data below). The lowest pH data show that at pH 4.0, where the cytosine pairs are more completely protonated, the addition of glycerol or low molecular weight PEG (e.g., PEG_200_) destabilizes the i-motif significantly (Δ*T_m_* ≈ −9 °C for PEG_200_). This destabilization is due to an increase in the electrostatic repulsion between charged cytosines, (Cyt-H^+^/H^+^-Cyt), brought about by the decrease in the dielectric constant for the water/PEG mixed solvent. The increase in i-motif stabilization at pH values above 4.5 exhibits a trend in that the stabilization of the i-motif is increased as the molecular weight of the PEG is increased, a clear indication of crowding due to the excluded volume effect of the larger polymers. In all of these solutions, there is a balancing act going on between the effect of the lowered dielectric constant (the same for all PEGs) and the effect of molecular crowding (increasing with PEG molecular weight). Sugimoto’s group reported the same effect, an increase in i-motif melting temperature with higher molecular weight PEG co-solutes [50].

The stabilization (and/or stability) of the c-MYC i-motif as a function of crowding agent concentration was again evaluated in DSC experiments. The melting (unfolding) temperature, *T_m_*, for the mutant c-MYC P1 promoter sequence i-motif without added PEG, and the increases in the *T_m_*s, Δ*T_m_*s, for the PEG_8000_ containing solutions are reported in Table 2. These experiments were done at PEG concentrations of 20%, 30%, and 40% and at pH values 4.5, 5.0, 5.5, 6.0 and 6.5.

**Table 2:** Change in the c-MYC promoter sequence i-motif melting temperature, *T_m_*, as a function of pH and concentration of added PEG_8000_.

	*T_m_* (°C)	Δ*T_m_* = *T_m_*(PEG_8000_) − *T_m_*(BPES) *		
pH	BPES	20%	30%	40%
4.5	45.6 ± 0.2	5.3	7.8	12.8
5.0	41.5 ± 0.2	8.8	11.0	15.8
5.5	33.4 ± 0.2	12.8	16.4	25.3
6.0	23.0 ± 0.2	9.5	19.3	27.4
6.5	11.2 ± 0.2 ^‡^	6.5 ^‡^	27.0	26.7

* The uncertainty in Δ*T_m_* is approximately ± 0.4 °C. ^‡^ *T_m_* and Δ*T_m_* melting temperature below 20 °C.

The data presented in Table 2 indicate significant increases in the *T_m_*s for the mutant human c-MYC i-motifs, particularly at higher pH values (e.g., pH = 6.5) in the presence of added PEG_8000_. The *T_m_*s reported in Table 2 are for the melting of the i-motif in the crowded environment and are not for disassociation or unfolding of the higher melting DNA/PEG complex. Increased stability of the i-motif, for example in solutions containing 30% or 40% PEG_8000_, results in a 20 to 27 °C increase in *T_m_* at pH 6.0 or 6.5. This is a clear indication that the i-motif is stabilized in a crowded solution environment at pH values approaching neutral pH and that the i-motif could persist in the crowded nuclear environment.

**Figure 3:**
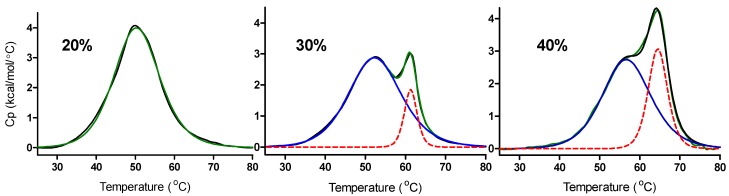
DSC melting curves for the thermal denaturation of the DNA species formed by the mutant c-MYC promoter sequence at pH 5.0 in 20%, 30%, or 40% w/w PEG_8000_ containing solutions.

Typical DSC melting curves observed for the thermal unfolding of the c-MYC C-rich promoter sequence oligonucleotide obtained at pH = 5.0 in 20%, 30% and 40% w/w PEG_8000_ containing solutions are shown in Figure 3.

The raw DSC data (black lines in Figure 3) have been fit to either one or two independent “two-state processes”. The DSC data for the 20% PEG_8000_ experiment (fit to a single two-state transition) shows only the melting of the i-motif. The DSC data for the 30% and 40% PEG_8000_ experiments (fit for two overlapping two-state transitions) show two separate effects upon the addition of PEG: 1) an increase in the *T_m_* for melting the i-motif (lower melting peak in these thermograms) and 2) a second peak with a higher melting temperature which is indicative of the presence of a small amount of a non-specific PEG/DNA complex. The blue solid lines and the red dotted lines in Figure 3 are deconvoluted components of the overall melting curves. The green lines in Figure 3 are the best fit lines, wherein the raw data have been modeled as either one or two separate and independent two-state melting process. The lower melting process, *T_m_s* ≈ 50, 53, and 57 °C, are attributed to the unfolding of the i-motif in these solutions while the higher melting process, *T_m_s* ≈ 62 and 65 °C are speculated to be the unfolding of a non-specific DNA/PEG complex formed in the 30% and 40% PEG_8000_ solutions. The assignment of the higher temperature melting to the DNA/PEG complex is based on its *T_m_* values which are much higher than *T_m_* values reported for unfolding of the c-MYC intramolecular i-motif [19,51].

**Figure 4:**
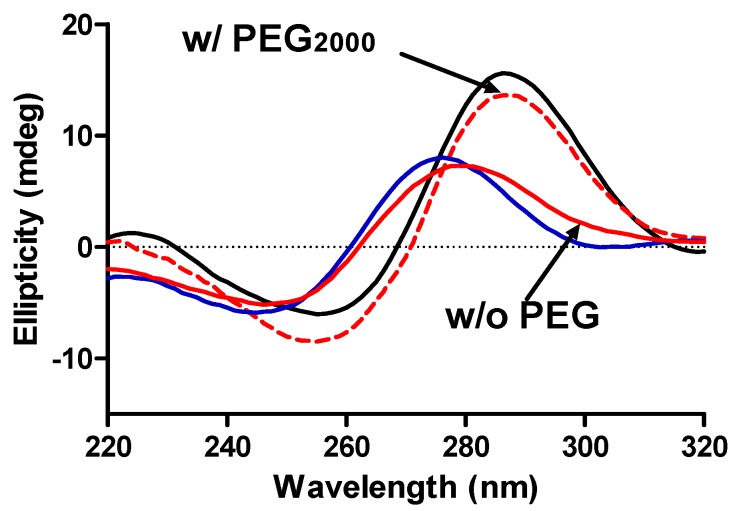
CD spectra of the c-MYC mutant promoter sequence i-motif DNA construct in BPES buffer at pH 4.5 (black ─), pH 6.0 (red ─) and pH 7.0 (blue ─) and with added PEG_2000_ (20% w/w) at pH 6.0 (red - - -).

CD experiments were used to establish the presence of the i-motif DNA structure in solution. The characteristic CD spectrum for a classical i-motif DNA exhibits a maximum ellipticity at (or near) 286 nm while the “i-motif-like” DNA structure exhibits a maximum ellipticity at (or near) 275 nm. CD spectra obtained for the mutant c-MYC i-motif in solutions with and without added glycerol are shown in Supplemental Figure S1. The characteristic i-motif spectra are observed in glycerol containing solutions at pH values of 4.0 to 5.5. At pH 6.0, the CD spectrum in glycerol is a mixture of the characteristic i-motif spectrum and the “*i-motif like*” spectrum observed at higher pH values. The CD spectra shown in Figure 4 are for solutions of the mutant c-MYC P1 promoter sequence i-motif in BPES buffer having pH values of 4.5, 6.0, and 7.0 and for a pH 6.0 solution containing 20% w/w PEG_2000_.

The CD spectrum at pH 4.5 in BPES buffer without added PEG is consistent with previously published reports in that the classical i-motif structure is observed in acidic solutions. The classic CD spectrum for the i-motif is shown as the black line in Figure 4 while the i-motif like spectrum, observed at pH > 6.0, is shown as the red line in Figure 4. A CD spectrum for the c-MYC oligonucleotide at pH = 7.0 is also shown (blue line) for comparison in Figure 4. It is clear that the classical i-motif structure is lost at pH 6.0 and higher; however, the addition of 20% PEG_2000_ drives the refolding of the i-motif into the classical structure at pH = 6.0. The addition of a molecular crowding agent, e.g., high molecular weight PEG, is apparently driving the folding/unfolding equilibrium toward formation of the more compact i-motif structure even though the cytosine pairs at this pH (*i.e.*, pH ≥ 6.0) are largely deprotonated.

**Figure 5:**
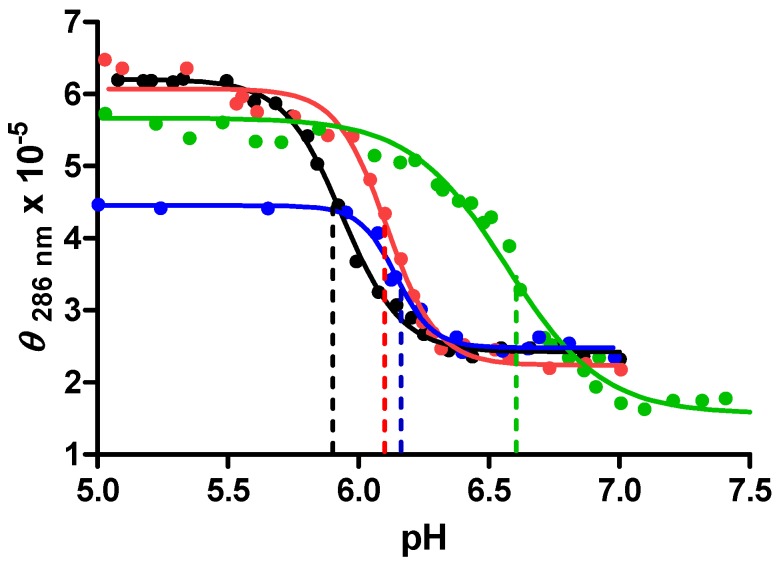
A plot of molar ellipticity at 286 nm *vs.* pH for each base titration of the mutant c-MYC promoter sequence in BPES buffer solution containing 0% (black ─/●), 20% (red ─/●), 30% (green ─/●) and 40% (blue ─/●) PEG_8000_.

The stabilization (and/or stability) of the c-MYC i-motif as a function of crowding agent concentration was evaluated in CD experiments. A plot of the molar ellipticity at 286 nm *vs.* pH for the c-MYC promoter sequence oligonucleotide in 0%, 20%, 30% or 40% PEG_8000_ is shown in Figure 5.

The CD data, molar ellipticities at 286 nm, used to make this plot suggest that the DNA exists as an i-motif structure in all of these solutions, at least at pH values below 6.0. The inflection point in each titration indicates the highest pH value at which the i-motif structure is the predominate folded DNA species in solution. The highest stable pH values for the 0%, 20%, 30% and 40% PEG solutions are 6.0, 6.1, 6.7, and 6.2 respectively. The maximum stabilization of the i-motif structure at pH 6.7 is observed in both the CD experiments shown in Figure 5 and in DSC measured increases in *T_m_* (shown in Table 2 above). The data in Figure 5 also shows that in both 30% and 40% w/w PEG_8000_ solution, the apparent molar ellipticity is decreased. This result is explained by a reduction in the free i-motif concentration in these solutions due to the formation of non-specific DNA/PEG complexes. DNA/PEG complex formation was also observed in the DSC data shown in Figure 3. In effect, as the PEG_8000_ concentration is increased to 30% or more, the formation of DNA/PEG complexes effectively reduces the i-motif DNA concentration by shifting the i-motif folding/unfolding equilibrium toward more unfolded DNA and less i-motif.

**Figure 6:**
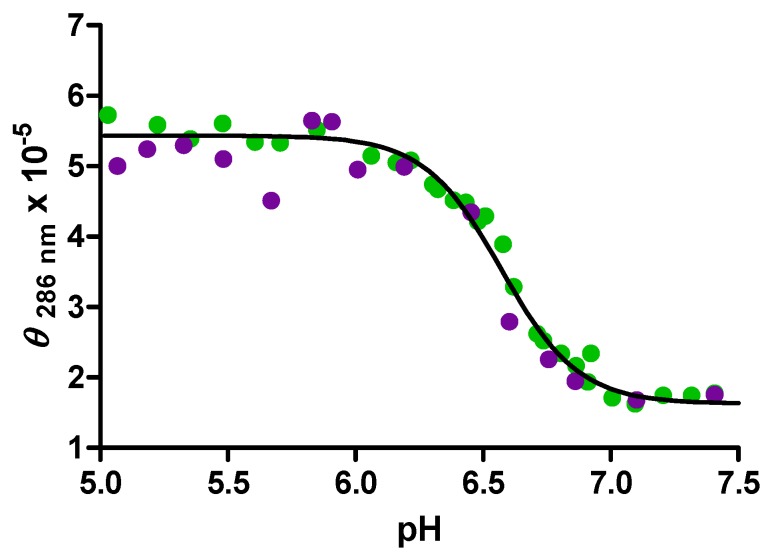
DNA molar ellipticity at 286 nm *vs.* pH for the acid or base titration of the DNA structure formed by the mutant human c-MYC promoter sequence in 30% PEG_8000_. The points shown in green (●) are for the forward titration in which NaOH was added to increase the starting pH of 5.0 up to the ending pH of 7.5. The points shown in purple (●) are for the reverse titration in which HCl was added to decrease the starting pH of 7.5 down to the ending pH of 5.0. The black line represents a global fit of both the forward and reverse titration data.

In order to determine whether the pH titrations were reversible, and to learn something about the kinetics of the folding/unfolding process in the presence or absence of molecular crowding agents, the titrations shown in Figure 5 were repeated in both directions, *i.e.*, going from low to high pH by the addition of NaOH and going from high pH to low pH by the addition of HCl. One of these cyclic pH titrations is shown in Figure 6.

No hysteresis observed in these CD titrations, in other words the acid and base titrations are virtually super-imposable. Both the forward and reverse titration agree with the experimental data shown in Figure 5 and with the expected result that the c-MYC i-motif is stable in these solutions to pH = 6.7. Within the response time of these CD titration experiments (<60 s), the folding/unfolding appears to be completely reversible, and there is no evidence that these systems are not at equilibrium.

**Figure 7:**
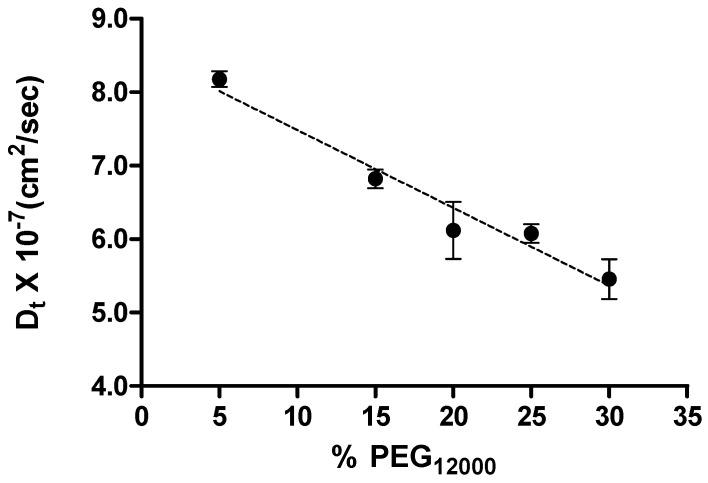
A plot showing the dependence of translation diffusion coefficient on concentration of PEG_12000_. The dash line represents the linear best fit and the slope of the line is the DNA-PEG interaction parameter.

In a further attempt to probe the non-specific interactions between the c-MYC promoter sequence oligonucleotide and higher molecular weight PEGs, we performed DLS experiments in the presence of PEG_12000_ where the non-specific complexation was expected to be even more prevalent than for the lower molecular weight PEGs. (CD and DSC data for the c-MYC i-motif in the presence of added PEG_12000_ are also shown in Table S1 and Figure S2 of the supplemental information.) The concentration of PEG_12000_ was varied in these experiments from 5% to 30% and the DLS data used to estimate the DNA translational diffusion coefficient, D_t_, as a function of PEG concentration. The DNA translational diffusion coefficients, D_t_s_,_ measured at PEG_12000_ concentrations 5% to 30% are plotted in Figure 7.

A simple linear relationship was observed for the DNA translational diffusion coefficient relative to the PEG_12000_ concentration. The negative slope for the Dt data plotted in Figure 7 is indicative of the attractive forces between the DNA and the crowding agent, PEG_12000_ [52], and further evidence for DNA/PEG complex formation.

Interestingly, DNA/PEG complexes have been reported in the literature as part of a biological technique that has been used to transport transgenic DNA across cell membranes [53,54,55,56,57]. DNA/PEG complexes were reported by Gietz *et al*. in which they introduced DNA into solutions containing 50% w/w PEG having a molecular weight of only 3,350 g/mol [53]. As mentioned earlier, Sugimoto’s group employed PEGs having molecular weights of 200 g/mol and 8,000 g/mol [50]. They reported i-motif stabilization to pH values above 7.0 and they attributed the stabilization to a shift of the cytosine N3 p*K*a from 4.8 to near 7.0 in the microenvironment provided in an i-motif formed in 20% PEG_8000_. This proposed shift in the cytosine p*K*a, δp*K*a ≈ 2.2, cannot be explained on the basis of the change in the effective dielectric constant. A change in the p*K*a of the cytosine N3 group from 4.8 to pH 7.0 would require a change in the dielectric constant from 78.6 (water at 298 K) to less than 14 as calculated from the Born approximation [58]. However, the measured dielectric constant for a 20% PEG/water solution has been reported to have a value of 53.8, much larger than the ε ≤ 14 calculated from the Born approximation [58,59]. Clearly, the dielectric constant for the 20% PEG solution used in Sugimoto’s experiments is not depressed enough to account for the dramatic change in the cytosine p*K*a suggested by Sugimoto [50].

A more recent publication by Buscaglia *et al.* describes the stabilization of the human telomere G-quadruplex in PEG solutions to be solely the result of the non-specific interactions driving DNA/PEG complex formation [60]. They were able to show that the quadruplex structure was unaffected by osmotic stress but that in the presence of the higher molecular weight PEGs, the non-specific complex formation influenced the folding equilibrium between the anti-parallel and parallel G-quadruplexes. The most dramatic effects were observed for PEG_8000_ and PEG_10000_ and at concentrations up to 42.5% (v/v). Although Buscaglia *et al.* discount molecular crowding in their study [60], we observed significant i-motif stabilization in solutions where there is no evidence of DNA/PEG complexation (e.g., 20% PEG_2000_, 20% PEG_4000_, and 20% PEG_8000_), at least by DSC.

## 3. Experimental

The mutant human c-MYC promoter i-motif forming sequence used in this study was designed based on the results of previous research. Previously we used a mutant sequence, Mut1, in which there were three **C**→**T** mutations and two **C**→**A** mutations. The purpose of these mutations was to simplify the folding and hopefully end up with a c-MYC promoter sequence that exhibited only a single i-motif folded conformer. The WT sequence has multiple repeated cytosines, which can interact to form more than one i-motif or “i-motif like” structure. In order to simplify the model, we designed Mut1 with the above listed 5 mutations and MutA with seven point mutations, including two additional **C**→**T** mutations. The sequences for the 5-base and 7-base mutants are listed in Table 3. Previous results indicate that there are three different structures formed by the five mutant sequence [19], while only a single structure is formed by the melting of the seven mutant sequence (data not shown).

**Table 3:** The wild-type (WT) 23mer human c-MYC promoter i-motif forming sequence and its two mutant sequences: Mut1 (5 mutations, shown in red) and MutA (7 mutations, shown in red).

	5′	1	2	3	4	5	6	7	8	9	10	11	12	13	14	15	16	17	18	19	20	21	22	23	3′
WT		C	T	T	C	C	C	C	A	C	C	C	T	C	C	C	C	A	C	C	C	T	C	C	
Mut1		C	T	T	T	C	C	T	A	C	C	C	T	C	C	C	T	A	C	C	C	T	A	A	
MutA		T	T	T	T	C	C	T	A	C	C	C	T	T	C	C	T	A	C	C	C	T	A	A	

The lyophilized oligonucleotide for MutA was purchased from Midland Reagent, Inc. (Midland, TX, USA). The oligonucleotide was dissolved in 1 mL of each buffer to make a stock DNA solution. DNA concentrations in the stock solution were verified using a Olis HP 8452A Diode Array Spectrophotometer (Bogart, GA, USA) and a molar extinction coefficient ε_260_ = 198,200 M^−1^cm^−1^.

All chemicals used in this study were purchased from Sigma-Aldrich (St. Louis, MO, USA) and used without further purification. The BPES buffer used in this study was prepared using 10 mM KH_2_PO_4_, 10 mM K_2_HPO_4_, 100 mM KCl, and 1 mM EDTA (for one liter of buffer). The solutions were filtered using a 500 mL Corning (Tewksbury, MA, USA) bottle top filter immediately after being prepared. The pH values of each of the buffer solutions were adjusted to the correct pH by adding either NaOH or HCl and measured using an Accumet XL15 pH meter (Fisher Scientific, Waltham, MA, USA). BPES buffer solutions containing glycerol, and PEG at various molecule weights and concentrations were prepared by measuring the mass of certain amount of BPES buffer, then the mass of co-solute was added based on the desired concentration and adjusted to desired pH values by adding either NaOH or HCl.

DSC experiments were performed on a Microcal VP-DSC (Piscataway, NJ, USA). The nominal concentration for DSC experiments was 300 μM. The DSC experiments were done over a temperature range from 10–90 °C at a scan rate of 90 °C/h. At minimal of three scans for each DSC experiment were acquired in order to ensure reproducibility. The data was analyzed with Origin 7 (Microcal, St. Louis, MO, USA) in order to determine the melting temperature (*T*_m_) and the enthalpy change, ΔH, for the unfolding process for every DNA sample.

CD experiments were performed on an Olis DSM 20 CD spectropolarimeter (Bogart, GA, USA). The CD samples were extracted from the post-DSC samples, which are collected after each DSC experiment. The nominal concentration for each CD sample was 3 μM. In some experiments (PEG_8000_ and PEG_12000_ CD titration experiments), CD samples were also made up from the pre-DSC samples. The CD titration samples were extracted from the leftover pre-DSC samples for each PEG_8000_ containing buffer. The pH of each pre-DSC sample was corrected to 4.0 before making the CD samples. The nominal concentration for each CD sample was 3 μM. The CD spectra were collected at 20 °C over the wavelength range of 200 to 325 nm with constant N_2_ gas flow. Data was analyzed using Olis GlobalWorks and GraphPad Prism 5 (La Jolla, CA, USA).

The CD titration samples had an initial pH value around 4.0. NaOH was added between scans to adjust the pH values gradually from 4.0 to 6.5 or higher. An Accumet flexible stem pH meter Microprobes was used to detect the pH value of the CD sample for each scan. The mixing time between scans were under 2 min. Data was also analyzed by using Olis GlobalWorks and GraphPad Prism 5.

Dynamic light scattering experiments were performed using a DynaPro^TM^ NanoStar (Wyatt Technology, Santa Barbara, CA, USA). Samples were prepared for the DLS measurements by diluting the stock DNA solutions to a final same concentration of 3 µM ELP in BPES buffer with and without added PEG_12000_. These sample solutions were stored at 4 °C, and then immediately before a DLS measurement, 1.5 mL of the dilute solution was placed in a conical tube and spun for approximately 5 min using a TOMY HF-120 Capsulefuge (CS Bio Co., Menlo Park, CA, USA) with an RCF of approximately 2000 G. Approximately 200 µL of the ELP sample was withdrawn from the conical centrifuge tube and used to overfill the 10 µL DLS quartz cuvette. All of these operations were done at room temperature. The DLS quartz cuvette was examined to ensure that no air bubbles were present in the active volume, and the cell allowed to equilibrate for 5 min after being placed into the DLS instrument. All DLS data was collected at 658 nm using a 10 s acquisition time. Sample collections were repeated for at least five times with five min intervals in between to ensure reproducibility. The laser power was set to 50%, the auto attenuation mode was disabled, and the translational diffusion value, D_t_, were recorded. The data was analyzed using the DYNAMICS software package (v. 7.1.0, Wyatt Technology, Santa Barbara, CA, USA) included with the instrument.

## 4. Conclusions

In this work, we probed the influence of both low molecular weight co-solutes and high molecular weight crowding agents on the stability of the c-MYC C-rich promoter sequence i-motif in crowded environments at pH values approaching neutral pH. Low molecular weight co-solutes included glycerol and 200 and 400 g/mol PEG. The crowded environment of the nucleus was simulated by higher molecular weight PEGs at concentrations as high as 40% w/w. We were able to demonstrate that the smaller co-solutes had the effect of improving charge stabilization through both a reduction in the water activity and a decrease in solvent dielectric properties.

Although not measured here, the volume of folded i-motif is estimated to be smaller than the volume of the DNA random coil. In effect, the i-motif to coil transition requires greater energy in these crowded solutions to unfold the DNA than would be needed in water. We started this work with the idea of answering just one question, can an i-motif exist as a stable structure at neutral pH. In the presence of 30% w/w PEG_8000,_ the i-motif was found to be stable in solutions having pH values as high as 6.7, fairly close the pH = 7.0 we were trying to achieve. We found that stabilization of the DNA i-motif by PEG, especially for higher molecular weight PEGs (8,000 to 12,000 g/mol), was offset to some degree by the non-specific interactions between the DNA random coil and the higher molecular weight PEGs. The best result we achieved, in terms of i-motif stabilization, was for the mutant c-MYC C-rich promoter sequence in 30% w/w PEG_8000_ solution where a stable i-motif structure was observed at pH values as high as 6.7. In these solutions, the i-motif still had a characteristic CD signal and a melting temperature in excess of 38 °C. Unfortunately, once the concentration of PEG_8000_ is increased to 40% w/w or more, or the PEG molecular weight is increased beyond 8,000 g/mol, the formation of DNA/PEG complexes results in a decrease in the fraction of the DNA existing as an i-motif and a concomitant increase in the fraction of the DNA present in the form of a DNA random coil complexed with PEG.

Nevertheless, we predict that stable i-motif structures can exist at neutral pH under the crowded solution conditions in the nucleus. It is clear from the data presented here that the stabilizing effects of low molecular co-solutes, *i.e.*, their effect on water activity and solvent dielectric constant, contribute only negligibly to i-motif stabilization at higher pH values. It is also clear that the formation of non-specific random coil DNA/PEG complexes would appear as destabilizing in terms of competing with the formation of i-motif DNA species. In comparison, the molecular crowding (or excluded volume effects) of higher the molecular weight co-solutes (e.g., PEG_4000–12000_) has been shown to contribute significantly to the stability of the more compact i-motif structure relative to the larger random coil DNA. Even though we achieved stabilization of the i-motif to pH = 6.7, we found that the use of the uncharged high molecular weight PEGs to model the crowded nuclear environment is problematic since these polymers associate with random coil DNA by hydrogen bonding [54]. Although we have not directly proven the existence of a stable c-MYC (or other oncogene) i-motif under physiological conditions, it seems that at this time, continued research into targeting the i-motif, remains a viable and novel approach to cancer treatment.

## Supplementary Materials

Supplementary Materials include CD spectra as a function of pH for the c-MYC i-motif in the presence of 20% glycerol, CD spectra as a function of pH in the presence of PEG_12,000_, and a table of DSC determined melting temperatures for the c-MYC i-motif in 10%, 20%, and 30% PEG_12,000_ that are not shown in the manuscript. These materials can be accessed at: http://www.mdpi.com/1420-3049/18/10/12751/s1
