# Supplementary material for: The Effect of Molecular Crowding on the Stability of Human c-MYC Promoter Sequence I-Motif at Neutral pH
Source: Molecules. 2013 Oct 15;18(10):12751–67. doi: 10.3390/molecules181012751 (PMC6270392; doi:10.3390/molecules181012751)
Supplement: Supplementary file 1 [file molecules-18-12751-s001.pdf]

Supplementary Materials

Figure S1. CD spectra for the c-MYC mutant promoter sequence i-motif DNA construct in BPES buffer with added glycerol (20% w/w). Red line (—): the CD spectra of the DNA structure at pH 4.0. Blue line (—): the CD spectra of the DNA structure at pH 4.5. Purple line (—): the CD spectra of the DNA structure at pH 5.0. Yellow line (—): the CD spectra of the DNA structure at pH 5.5. Green line (—): the CD spectra of the DNA structure at pH 6.0. Orange line (—): the CD spectra of the DNA structure at pH 6.5; Black line (—): the CD spectra of the DNA structure at pH 7.0.

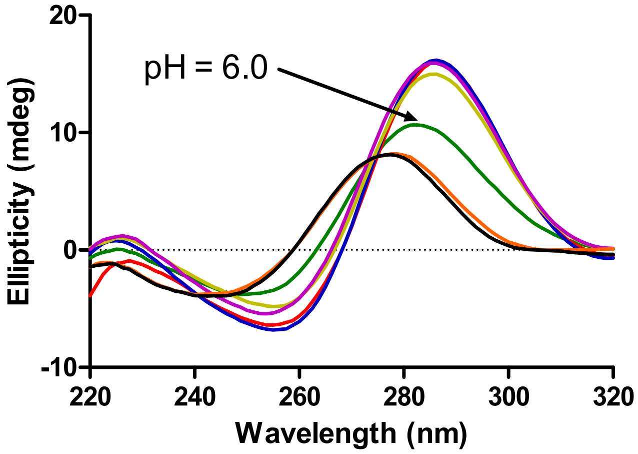

Results of the DSC melting experiments for the mutant c-MYC i-motif forming sequence in 0%, 10%, 20% and 30% w/w PEG_{12000} containing buffer solutions at pH 5.0 and 6.0 are summarized in Table S1. The DSC results show that at both pH 5.0 and 6.0, the stability of the i-motif, in terms of melting temperature, increases as the concentration of PEG_{12000} increases. This observation is consistent with the results of the i-motif stability in PEG_{8000} containing buffers with PEG concentrations from 0% to 30%.

Table S1. The DSC determined melting temperatures, T_ms of the i-motif structure formed in 0%, 10%, 20% and 30% w/w PEG_{12000} solutions at pH 5.0 and 6.0.

% PEG_{12000}	T_m of oligonucleotide in pH 5.0 solutions	T_m of oligonucleotide in pH 6.0 solutions
0	41.5	23.0
10	44.9	26.4
20	48.5	36.8
30	51.0	46.7

If we compare the T_ms of the c-MYC i-motif in two different PEG containing buffers (PEG_{8000} and PEG_{12000}), but having the same concentration and the same pH values, we see that at the same concentration (20% or 30% w/w), PEG_{8000} exhibits the better stabilization of the i-motif in pH 5.0 solutions, while PEG_{12000} exhibits the better stabilization of the i-motif at pH 6.0. The T_m data in Table 2 in combination with the T_m data in Table S1 show that at pH = 5.0, the melting of the c-MYC i-motif occurs 1.5 to 2.1˚C lower in 20% PEG_{12000} solutions than in 20% PEG_{8000} solutions. In contrast, at pH = 6.0, the melting of the c-MYC i-motif occurs approximately 4˚C higher in 20% PEG_{12000} solutions than in 20% PEG_{8000} solutions. All of the DSC melting experiments done in the

PEG_{12000} show the presence of a second higher melting peak in the thermogram that we have attributed to the disassociation or unfolding of a DNA/PEG complex (see Figure 3).

CD spectra for pH titration experiments done on the mutant c-MYC promoter sequence in 0%–30% w/w PEG_{12000} containing buffer solutions are shown in Figure S2. The CD results show that in 10%, 20% and 30% PEG_{12000} solutions, the highest pH at which a stable i-motif occurs in each of these solutions is approximately pH = 6.1, pH = 6.2, or pH = 6.3. Again, these results in terms of i-motif stabilization as a function of PEG concentration are similar for PEG_{8000} and PEG_{12000}.

Figure S2. CD spectra for pH titration experiments done for the mutant c-MYC promoter sequence in 0-30% w/w PEG_{12000} containing buffer solutions.

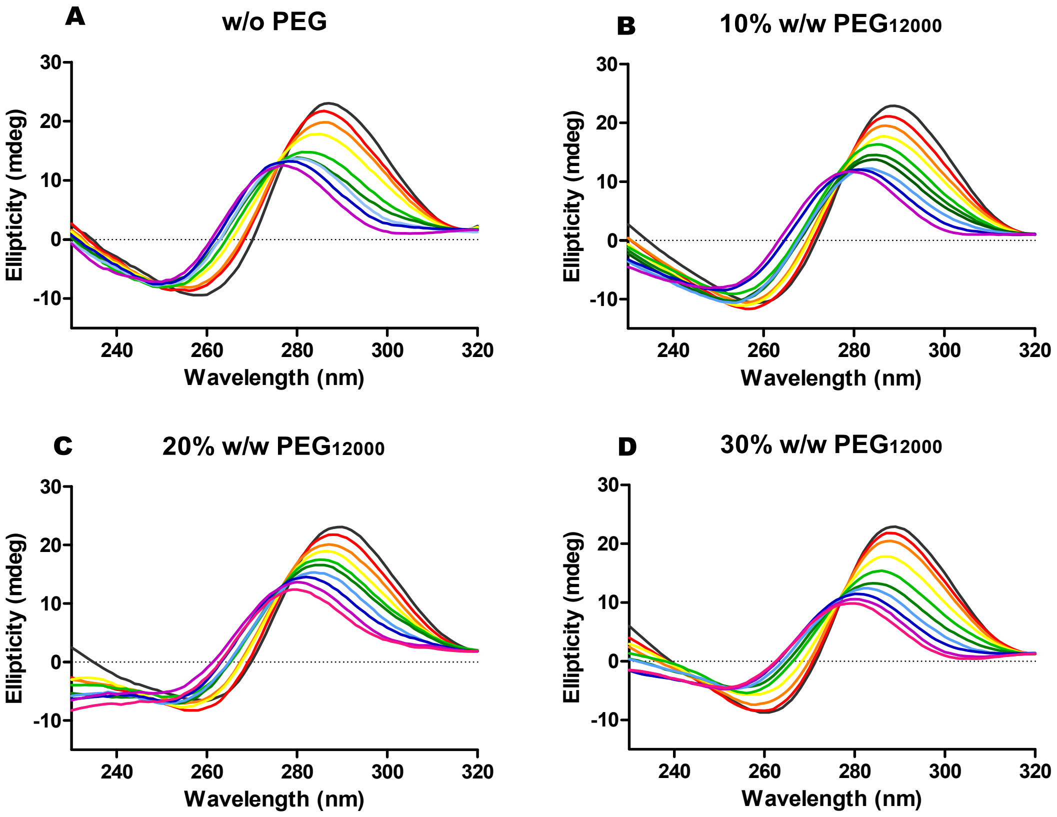

	pH value								
	Black	Red	Orange	Yellow	Light green	Dark green	Light blue	Dark blue	Purple
Panel A	4.50	5.60	5.75	5.81	5.84	5.92	5.99	6.08	6.21
Panel B	4.09	5.75	5.90	5.95	6.06	6.11	6.17	6.29	7.00
Panel C	4.05	5.81	6.00	6.05	6.14	6.22	6.30	6.45	6.72
Panel D	4.93	5.79	6.03	6.16	6.25	6.34	6.39	6.47	6.91
